# Editorial: Thoracic outlet compression, thoracic outlet syndrome, with or without complications or sequelae: mechanisms, investigations and treatments

**DOI:** 10.3389/fphys.2023.1334668

**Published:** 2023-11-28

**Authors:** Pierre Abraham, Paul W. Wennberg, Lianrui Guo

**Affiliations:** ^1^ Department of Physiology, Université d’Angers, Angers, France; ^2^ Mayo Clinic, Rochester, MN, United States; ^3^ Xuanwu Hospital, Capital Medical University, Beijing, China

**Keywords:** thoracic outlet, syndrome, compression, complications, pain, movement, neurogenic, vascular

The characteristics of the human shoulder and, specifically, the small diameter of the pathway from the chest to the upper limb predispose the neurovascular bundle to external compressions by adjacent structures at different levels of the thoracic outlet during upper limb movements (mostly during abduction). There are a large number of publications (more than 40 review papers in the last 2 years) from diverse specialties (including radiology, vascular medicine, surgery, sports medicine, rehabilitation, and neurology) on physiopathology, etiology, epidemiology, non-invasive diagnosis, invasive imaging, complications, and medical or surgical treatments (Daley et al., 2022; Al-Redouan et al., 2023; Hoexum et al., 2023; Perdikakis et al., 2023; Teijink et al., 2023). This large number of publications illustrates the fact that many questions remain unsolved about this troublesome medical issue.

Defining TOS is not the least of the problems. We recently proposed that contrary to most publications, asymptomatic compression should not be referred to as thoracic outlet syndrome (TOS) but rather as thoracic outlet compressions (TOC), while “thoracic outlet compression with complications or sequelae” would be called TOX (Abraham et al., 2023). In this perspective, the original case reported by Mansouri and Lutz is fascinating: how should this case be called? The external compression is obvious, the mechanism of which does not result from normal anatomic structures but very likely by the edema induced by the initial surgery, which might explain the long-term spontaneous favorable evolution. The symptoms are present, but McCleery syndrome is generally considered a chronic phenomenon, while here, the venous outflow impairment seems to be an acute and transient event. Nevertheless, the positional characteristics that are almost systematically described by the patients during TOS of either neural or vascular origin are not present (or not reported). With this missing positional characteristic, this case should probably not be referred to as TOS. Since is it symptomatic, it is not TOC. Last, although the venous outflow impairment occurred as a complication of surgery, there is no complication resulting from the outflow impairment (absence of thromboses), meaning it is neither TOX.

The experience of Davies and Hart in patients with arteriovenous fistula for hemodialysis is also fascinating. Indeed, while TOS is mainly observed in relatively young adults with few if any co-morbid conditions (except for the osteo-tendineous lesions or atypical anatomies that participate in the narrowing of the outlet space), this series is of major importance, specifically in perspective of the number of subjects in the general population that have a compression during positional maneuvers. Should venous compression be systematically searched before creating an arterio-venous fistula? If so, how? While the majority of studies published to date deal with treatment of TOS, there is a call for action to provide as many accurate and adapted investigations as possible to try to fill the gap between the presence of a positional TOC and the presence of positional symptoms. The two articles by Szaro et al. and Hersant et al. both illustrate, with different methodological approaches, that there is still a long way to go to standardize the results of investigations (here, with the RMI approach in neural TOS) and also that “new” tests may still emerge with relatively simple techniques to provide arguments for the relationship between symptoms and the positional compression (here, the presence of ischemia in arterial TOS). Contrary to a recent article claiming that ultrasound (Goeteyn et al., 2022) and EMG (Rousseff et al., 2005) are useless in patients suspected of TOS, we advocate that the holistic diagnosis of TOS can be supported by results of different investigations, with the advantage that consistent results can threaten the responsibility of TOC in the symptoms but with the risk that eventual inconsistencies add complexity to an already difficult diagnosis.

Nevertheless, providing additional arguments to support the coherence of the triad constituted by symptoms, compression, and positional characteristics is essential because the results from treatments are far from perfect, as shown by the two articles of de Kleijn et al.—who demonstrate symptom-free survival of 35.6%–48.1% depending on the treatment strategy in 115 patients treated over a period of 11 years for Paget–Schroetter syndrome—and de Kleijn et al.—who demonstrate 77.8%–81.8% depending on the presence or absence of complications in 20 patients treated over a period of 16 years for arterial TOS.
